# Patterns of Antibiotic Resistance Among Group B *Streptococcus* Isolates: 2001–2004

**DOI:** 10.1155/IDOG/2006/57492

**Published:** 2006-03-19

**Authors:** Lubna Chohan, Lisa M. Hollier, Karen Bishop, Charles C. Kilpatrick

**Affiliations:** Department of Obstetrics, Gynecology and Reproductive Sciences, University of Texas Medical School at Houston, Houston, TX 77030, USA

## Abstract

The objectives were to determine the prevalence of group B streptococcus (GBS) and to characterize antibiotic resistance patterns. All pregnant women presenting to the triage units at two urban hospitals during three intervals from 2001 to 2004 were included. Each interval lasted approximately four weeks. Swabs were inoculated into selective broth and cultured on tryptic soy agar with 5% sheep blood. GBS was identified using the StrepTex latex agglutination system. GBS positive cultures were tested for their resistance to ampicillin, erythromycin, clindamycin, and cefazolin. GBS was isolated from 154 (12.2%) of 1264 swabs collected during the study period. African-American women were more likely to be colonized with GBS than Caucasians and Hispanics. Resistance to routinely administered antibiotics was common, but there were no statistically significant increases in resistance to antibiotics over the study period. Ongoing surveillance of antibiotic resistance patterns is important in determining optimal prophylaxis and therapy.

## INTRODUCTION

Despite a substantial decline in the incidence of early onset group B *Streptococcus* (GBS) infections in the newborn, GBS remains an important cause of morbidity and mortality in the newborn period. Clinical trials have demonstrated the effectiveness of antibiotic prophylaxis administered during labor to women colonized with GBS in reducing disease in the newborn [1–3]. Protocols that incorporate routine screening for group B *Streptococcus* with intrapartum chemoprophylaxis have recently been shown to prevent more cases of early-onset disease than risk-based approaches [4].

One emerging threat to the current success of screening and prophylaxis regimens is the emergence of antibiotic resistance among group B *Streptococcus* strains. Penicillin G is the antibiotic of choice for prophylaxis [5]. Other options include ampicillin, and for penicillin-allergic patients, cefazolin, clindamycin, erythromycin, or vancomycin. In several studies, significant resistance to clindamycin and erythromycin has been identified [6–8].

Because of the importance of intrapartum antibiotics for prevention of invasive GBS disease in the neonate and the concern regarding emerging antibiotic resistance, the objectives of this study were to determine GBS prevalence by ethnicity as well as GBS antibiotic resistance patterns in our population.

## SUBJECTS AND METHODS

Any pregnant woman (greater than 20 weeks gestation) who presented to triage at Memorial Hermann Hospital (MHH) or Lyndon Baines Johnson General Hospital (LBJ) was eligible for the study. The study took place at three intervals between 2001–2004. Each interval lasted approximately four weeks in November 2001, November 2002, and January 2004. Administrative approval for the study was obtained from the institutional IRB and the IRB of both hospitals. All patients were included without regard to race, parity, or previous antibiotic use in pregnancy. In 2002 and 2004, additional information was obtained regarding patient's race and antibiotic use during the current pregnancy. All women were questioned about previous visits to triage during the study period, and repeat surveillance cultures were not performed.

In 2001, swabs were obtained from the vaginal sidewall. In 2002 and 2004, swabs were taken from the vaginal sidewall as well as anorectum. Transport swabs with liquid Stuart's medium (Fisherbrand, Houston, Tex ) were plated onto tryptic soy agar with 5% sheep blood (PML Microbiologicals, Wilsonville, Ore) and inoculated into selective broth (Lim broth, PML Microbiologicals, Wilsonville, Ore) and incubated for 18–24 hours. The broth was subcultured to plates of tryptic soy agar with 5% sheep blood (PML Microbiologicals, Wilsonville, Ore). Colonies characteristic of GBS were identified using the StrepTex latex agglutination system (Murex Biotech Limited, Kent, UK). To test for antimicrobial resistance, the disk diffusion method (Becton Dickinson, Sparks, Md) was used and the National Committee for Clinical Laboratory Standards guidelines were used to interpret the results. The antibiotics tested were: ampicillin 10 μg, erythromycin 15 μg, clindamycin 2 μg, and cefazolin 30 μg. Intermediate results were considered susceptible. Because zones of streptococcal growth inhibition to determine resistance are not available for cefazolin, we used values for *Staphylococci*.

All statistical calculations were performed with StataSE 8.2 (Stata Corp, College Station, Tex). Demographic characteristics were compared with chi-square and logistic regression. Changes in the proportion of GBS isolation and the proportion of resistant organisms over the study period were compared using the np trend test (a nonparametric extension of the Wilcoxon rank-sum test). The odds of GBS colonization were compared with logistic regression.

## RESULTS

A total of 1264 patients were screened during this study. Of these, 301 patients (24%) were screened in 2001, 446 patients (35%) in 2002, and 517 patients (41%) in 2004. Of the total 1264 patients, 989 (78%) were from LBJ and 275 (22%) were from MHH. The majority of participants were Hispanic (75%, 72%, resp, in 2002 and 2004), followed by African-American (18%, 20%, resp), Caucasian (6%, 6%), and other (1%, 2%).

**Figure 1:**
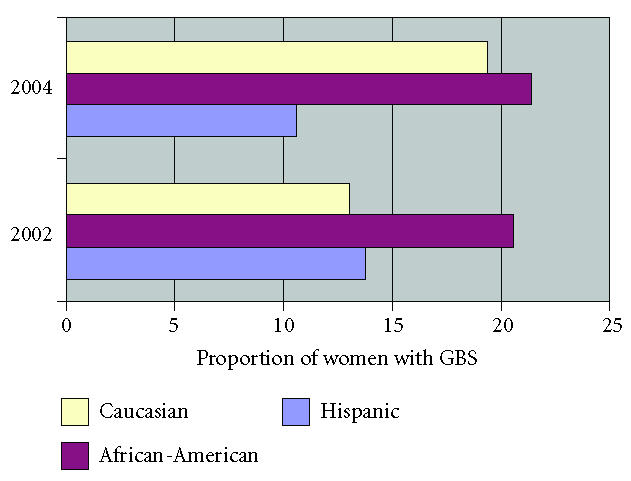
GBS isolation rates stratified by ethnicity and year.

Group B *Streptococcus* was found in 154 of 1264 (12.2%) swabs. Of the 154 swabs, 20 were obtained in 2001, 67 were obtained in 2002, and 67 were obtained in 2004. The proportion of women with GBS varied by ethnic background (Figure 1). African-American women were significantly more likely to be colonized with GBS (OR 1.9, 95% CI 1.2, 2.9) than were non–African-American women.

**Figure 2:**
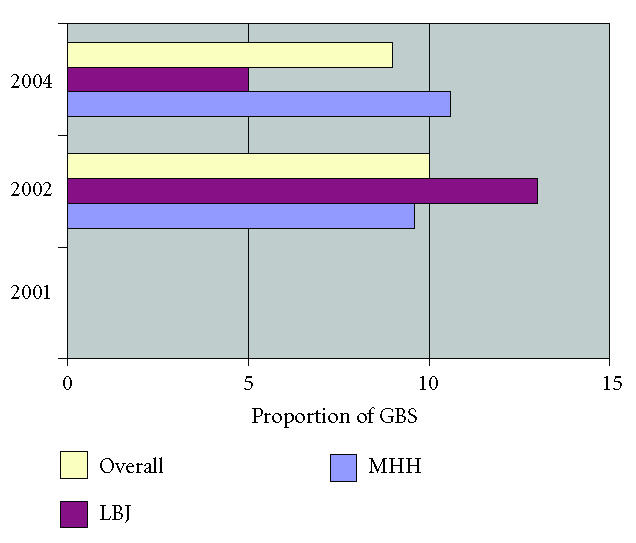
Resistance to erythromycin among GBS isolates stratified by year and location.

**Figure 3:**
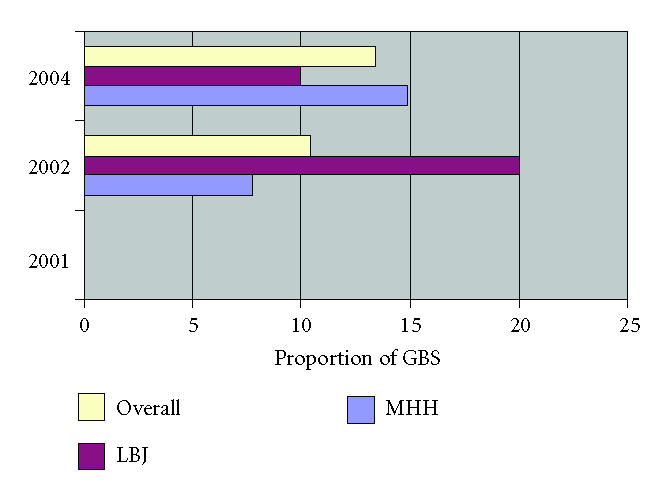
Resistance to clindamycin among GBS isolates stratified by year and location.

**Figure 4:**
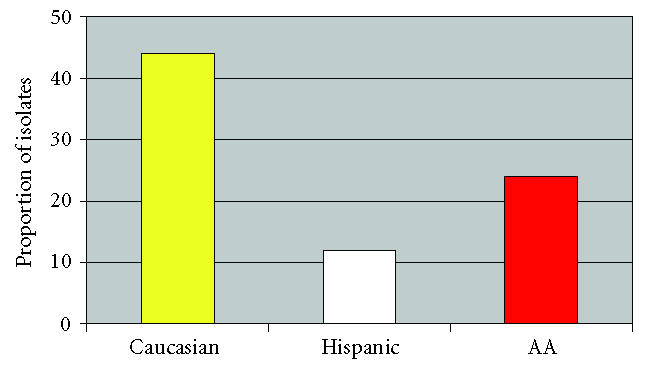
Antibiotic resistance stratified by ethnicity.

Of the 154 GBS isolates, none were resistant to ampicillin. Resistance to erythromycin was found in no isolates in 2001, 10% (7 of 67) in 2002, and 9% (6 of 67) in 2004, *P* = .41 (Figure 2). Resistance to clindamycin was found in no isolates in 2001, 10% (7 of 67) in 2002, and 13% (9 of 67) in 2004, *P* = .38 (Figure 3). Using zone of inhibition values for *Staphylococci*, resistance to cefazolin was found in no isolates in 2001, 2% (1 of 67) in 2002, and 4% (3 of 67) in 2004, *P* = .20. The prevalence of resistance among GBS isolates was nearly twice as high among Caucasian women (44%) compared to African-American (24%) or Hispanic women (12%), *P* = .01 (Figure 4). The proportion of women with GBS resistant to either clindamycin or erythromycin increased from 0% to 15% to 19% across the study period (*P* = .05).

We obtained antibiotic histories on our patients at the time of screening for the last two years. We found that 226/924 (25%) of the women screened had taken antibiotics during the pregnancy. The two most commonly used antibiotics were nitrofurantoin (7%) and flagyl (6%). Twenty-one (3%) used two or more different antibiotics. African-American women were twice as likely to report antibiotic use during pregnancy (OR 2.4, 95% CI 1.7, 3.4). There was no difference in the prevalence of group B *Streptococcus* isolation among women who had taken antibiotics during the pregnancy and those who had not (OR 1.1, 95% CI 0.7, 1.7). Antibiotic resistance was not significantly related to exposure to a particular antibiotic, but the sample sizes for these comparisons were small (data not shown).

## DISCUSSION

In this study of 1264 anorectal and vaginal swabs with 154 GBS isolates in women presenting to labor and delivery, resistance was found to antibiotics commonly used for GBS prophylaxis as well as for premature rupture of membranes. While antibiotic resistance varied by hospital and year, there was no statistically significant increase in antibiotic resistance over the study period.

Numerous other authors have evaluated the prevalence of antibiotic resistance among group B *Streptococcus* over the last 15 years [7–11]. In 1990, Berkowitz and colleagues evaluated 156 GBS isolates and found uniform sensitivity to penicillin and ampicillin and resistance to erythromycin, clindamycin, and cefoxitin in 3.2%, 2.5%, and 1.2% of strains, respectively, [12]. Among rectovaginal samples, studies performed more than 10 years later have found clindamycin resistance ranging from 3%–21% and erythromycin resistance ranging from 5%–29% [7–11, 13]. Among GBS isolates from neonatal sepsis, erythromycin resistance was present in 8% of strains, with 4.5% of these also resistant to clindamycin [9]. Simoes and colleagues used a similar testing method to evaluate the response of GBS to cefazolin and found 6%–8% of GBS isolates were not considered sensitive [14].

We found that GBS carriage was more common in African-American women, similar to previous reports [15, 16]. In Meyn's study of 1248 nonpregnant women, African-American race was associated with a higher prevalence of GBS at screening and also with an increased rate of acquiring vaginal GBS colonization [16]. This differential underscores an opportunity to improve the disparity in perinatal morbidity and mortality for African-Americans.

Manning and colleagues evaluated 117 group B *Streptococcus* isolates obtained between 8/1999 and 3/2000 in Michigan [17]. In their cohort, black ethnicity was associated with resistance. In contrast, in our study, Caucasian ethnicity was associated with carriage of a resistant organism. This was true in spite of the fact that African-American women were more likely to report antibiotic use during their pregnancy. In the Manning study and in our own, the ethnic group with increased carriage of resistant organisms had the smaller sample size. Further study with larger diverse populations, or multicenter national sampling studies will be necessary to determine the validity of the ethnic variations in carriage of resistant organisms.

With more widespread use of antibiotics, selection of antibiotic-resistant GBS may occur. If resistance continues to be identified and increasing, changes in our current practices will need to occur. Current recommendations include GBS susceptibility testing for clindamycin and erythromycin in penicillin-allergic patients [5]. Identification of factors associated with colonization and particularly colonization with resistant organisms is important in maintaining the success of current programs to reduce perinatal morbidity and mortality from invasive GBS disease. Ongoing surveillance of antibiotic resistance patterns in both pregnant women and their infants will be important in determining optimal prophylaxis and therapy for our patients.
